# Financial inclusion and households’ choice of solid waste disposal in Ghana

**DOI:** 10.1186/s12889-022-13512-2

**Published:** 2022-06-04

**Authors:** Mustapha Immurana, Kwame Godsway Kisseih, Mbanba Ziblilla Yakubu, Hadrat Mohammed Yusif

**Affiliations:** 1 Institute of Health Research, University of Health and Allied Sciences, Ho, Ghana; 2 Christian Health Association of Ghana Secretariat, Accra, Ghana; 3 Department of Applied Economics, University for Development Studies, Tamale, Ghana; 4 Department of Economics, Kwame Nkrumah University of Science and Technology, PMB, Kumasi, Ghana

**Keywords:** Solid waste disposal, Financial inclusion, Environmental health, Sanitation, Multinomial probit, Ghana

## Abstract

**Background:**

As the amount of solid waste generated by households in Ghana continues to grow, policy makers are preoccupied with finding better means of managing these solid wastes. To this end, a number of studies have been conducted on the factors that determine the choice of solid waste disposal method among households in Ghana. Notwithstanding, while financial inclusion is deemed as an effective tool for improved solid waste management, none of these studies paid attention to it. This study therefore, investigates the effect of financial inclusion on the choice of solid waste disposal method among households in Ghana.

**Methods:**

The study uses data from the Ghana Living Standards Survey round 7 (GLSS7). The multinomial probit regression is used as the empirical estimation technique.

**Results:**

Our results show that financial inclusion increases the likelihood of households opting for the collection method of solid waste disposal relative to burning, public dumping and indiscriminate disposal of solid waste.

**Conclusion:**

Financial inclusion enables households to opt for a healthy solid waste disposal method (collection method), hence, in policy makers’ attempts to improve solid waste disposal, paying attention to financial inclusion can be a very useful strategy.

## Introduction

Each year, at the global level, the municipal solid waste generated is 2.01 billion tonnes, and this is expected to increase to 3.40 billion tonnes by 2050 [1]. For sub-Saharan Africa (SSA), the growth in solid waste is even expected to more than triple by 2050 [1]. Nonetheless, globally, a minimum of 33% of waste is not managed in a manner that is environmentally safe [1]. For instance, in low-income countries, more than 90% of waste is openly burned or disposed in dumps that are unregulated [2]. These ways of waste disposal, have negative environmental, safety and health effects because, they can lead to climate change via the generation of methane, as well as serving as fertile grounds for disease vectors [1, 2]. While waste collection is a major step towards proper waste disposal, it is however, highly concentrated among high-income countries. For instance, while at least 90% of waste is collected in Europe and North America, in SSA, only 44% of waste is collected [1].

The situation in Ghana is not different. Estimates show that daily, 12,710 tonnes of household waste are generated, with an average of 0.47 kg per person [3]. However, only 10% of solid waste is disposed properly [4], with majority of them ending up in streams, open places and drains [5]. Given this, many cities and towns in Ghana are overwhelmed with how to properly manage solid waste [5]. With the population of Ghana exhibiting an increasing trend [6], devising means of ensuring proper waste management, cannot be overemphasised. However, to arrive at enhanced proper waste management, there is the need to know the factors that determine households’ choice of solid waste disposal method.

This has resulted in several studies examining issues of solid waste management in Ghana [3, 4, 7–21]. However, while financial inclusion (making individuals/firms have access to affordable and helpful financial services and products in a sustainable way [22]) is regarded as a major strategy that can be employed towards improving waste disposal [2], none of the above studies paid attention to it. Thus, financial inclusion has been found to enhance poverty alleviation, reduce income inequality as well as enhance economic growth and net wealth benefits, especially among poor people [23–26]. Therefore, since the theory of demand for health posits that the demand for health inputs (such as the collection method of solid waste disposal) would increase as income increases [27], a rise in income as a result of financial inclusion would enhance people’s ability to afford proper or healthy waste disposal methods (such as the collection method) which normally comes at a fee, all other things being equal.

This study, therefore investigates empirically, the effect of financial inclusion on the choice of solid waste disposal method by households in Ghana, making it the first of its kind to the best of our knowledge. The findings of the study help to reveal to stakeholders as to whether financial inclusion can be used as a strategy towards enhancing the use of healthy solid waste disposal methods among households in Ghana.

### Theoretical framework

**Fig. 1:**
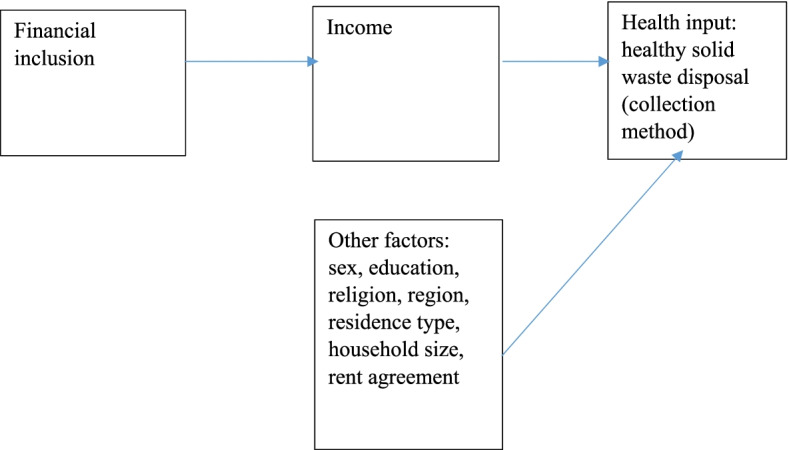
Theoretical framework of the link between financial inclusion and healthy solid waste disposal methods

This section provides the theoretical framework of the link between financial inclusion and healthy solid waste disposal methods  (Fig. 1) drawing on the theory of demand for health [27] and the vulnerable group theory of financial inclusion [28].

The theory of demand for health as indicated already postulates that, a rise in income would lead to an increase in the demand for health inputs such as healthy solid waste disposal methods, all other things being equal [27]. The vulnerable group theory states that financial inclusion programmes should target vulnerable groups such as the poor [28]. Thus, using these theories, a rise in income as a result of financial inclusion would increase the ability of people (especially vulnerable ones such as the poor) to afford healthy solid waste disposal methods.

## Methods

### Data and variables

Data for this study is sourced from the Ghana Living Standards Survey round 7 (GLSS7) which was carried out from October, 2016 to October, 2017. The survey used a two-stage stratified sampling technique. One thousand (1000) enumeration areas were used as the primary sampling units, which were further classified into rural and urban areas. A complete listing of households in the enumeration areas was done to get the secondary sampling units. In the second stage, 15 households were systematically selected from each of the enumeration areas, resulting in a sample size of 15,000 households. However, only 14,009 households were interviewed [29]. It must be stressed that not all variables have observations of up to 14,009.

The dependent variable in this study is the method of solid waste disposal among households (how households dispose their refuse). The options are collected (by local authorities or private firms or individuals), burning, public dump and dumped indiscriminately. Among these methods, as already indicated, collected is the healthiest or most proper solid waste disposal method. This is because, while burning can lead to the inhalation of pollutants by households and other community members, using public dump and indiscriminate dumping may result in the outbreak of diseases such as cholera. We therefore use the collection method of solid waste disposal (collected) as the control/base/reference category. Thus, for the purpose of this study, the collection method is referred to as proper or healthy solid waste disposal, hence, these terms are used interchangeably.

Financial inclusion (measured by whether the household head contributes to a loan or saving scheme or owns a bank account), is the main independent variable. This indicator, directly or indirectly, covers both the usage and access aspects of financial inclusion because, from the data, it can be seen that the household heads have access to different kinds of financial institutions (investment/mortgage/, commercial bank, rural/community bank, credit union, mobile money and susu (traditional savings and loans scheme)) and also use products/services such as Automated Teller Machine (ATM) cards, E-zwitch, electronic banking and loans. Using this indicator aids in comparing the choice of solid waste disposal methods among households with and without financial inclusion, hence, revealing the effect of financial inclusion on the choice of solid waste disposal method.

The remaining independent variables are religion, region, residence (urban or rural), age, sex and educational qualification of the household head, household size, total household wage income (income) and rent agreement. For the purpose of the present study, religion, educational qualification and rent agreement are recoded from their original nature. Apart from total household wage income, household size and age of the household head that are continuous, all the rest of the independent variables are categorical, so we treat them as dummy variables.

### Statistical analyses

The socio-demographic characteristics of the respondents are presented using descriptive statistics. We use the Pearson Chi2; a bivariate approach, to examine the association between each of the categorical independent variables and the choice of solid waste disposal method. However, bivariate analysis considers only two variables at a time without controlling for the effects of other variables that can affect the choice of solid waste disposal method. We therefore use the multinomial probit regression to examine the effect of financial inclusion on the choice of solid waste disposal method among households in Ghana, whiles controlling for all the other independent variables. This regression technique is used because of the inability of the Ordinary Least Square (OLS) estimator to capture the discrete nature of the dependent variable [30]. Moreover, we do not use the multinomial logit regression due to its assumption of ‘independence of irrelevant alternatives’ [31], which implies that, the choice of a solid waste disposal method, is independent of the existence of another. We therefore run two models, first, a model with the dependent and all the independent variables (Table 3(1)) and second, a model with all these variables and an interaction of financial inclusion with income (financial inclusion x income) (Table 3(2)). Using the interaction term is very important because, income is a major channel via which financial inclusion can affect the choice of solid waste disposal method. The observations of our regression results are less than 14,009 since the observations of some variables are not up to 14,009.

In order to avoid biasedness and also make our results nationally and regionally representative, all the analyses in this study are weighted as recommended by the Ghana Statistical Service and ICF [32]. We do so using the ‘iweight, svyset and svy’ routines in STATA. Thus, all analyses in this study are done using STATA 14.0.

## Results

In this section, the socio-demographic characteristics of respondents as well as the bivariate and multivariate (regression) results of the study are presented (Tables 1, 2 and 3).

**Table 1:** Socio-demographic characteristics of respondents

**Variable**			**%**
**Method of solid waste disposal**			
Collected			21.72
Burned by household			19.45
Public dump			47.96
Dumped indiscriminately			10.87
**Financial inclusion**			
Yes			55.99
No			44.01
**Sex **			
Male			66.62
Female			33.38
**Educational qualification**			
None			30.25
Yes			69.75
**Religion**			
No religion			6.13
Christian			74.10
Traditionalist/other			3.74
Islam			16.03
**Region**			
Western			10.32
Central			8.33
Greater Accra			18.14
Volta			7.50
Eastern			11.75
Ashanti			22.76
Brong-Ahafo			9.29
Northern			6.57
Upper East			3.11
Upper West			2.23
**Residence**			
Urban			56.02
Rural			43.98
**Rent agreement**			
Owning			42.16
Renting			27.56
Rent free			29.72
Perching/squatting			0.56
	Mean	Min	Max
**Income (in Ghana Cedis)**	3670.29	0	216,000
**Age (in years)**	46.24	15	99
**Household size**	4.20	1	28

Source: Authors’ computation from GLSS7

Total percentages are rounded as 100

**Table 2:** Bivariate analysis of solid waste disposal methods and categorical independent variables

Variable	Collected	Burned by household	Public dump	Dumped indiscriminately	Level of significance
**Financial inclusion**					
Yes	29.31	18.36	45.97	6.36	***
No	12.07	20.84	50.49	16.61	
**Sex **					
Male	21.59	21.19	44.80	12.42	***
Female	21.98	15.98	54.25	7.79	
**Educational qualification**					
None	13.34	20.63	54.66	11.38	***
Yes	29.63	17.62	47.26	5.50	
**Religion**					
No religion	10.19	28.84	46.52	14.46	***
Christian	23.82	18.49	49.73	7.95	
Traditionalist/other	4.13	40.68	18.66	36.52	
Islam	20.57	15.30	47.24	16.90	
**Region**					
Western	6.89	10.90	76.15	6.06	***
Central	7.32	18.80	68.20	5.68	
Greater Accra	65.49	14.17	17.62	2.72	
Volta	5.73	39.72	31.68	22.86	
Eastern	5.66	29.05	55.81	9.48	
Ashanti	27.73	11.45	57.46	3.36	
Brong-Ahafo	2.49	16.05	67.29	14.17	
Northern	6.03	23.28	33.16	37.53	
Upper East	8.07	52.59	7.07	32.27	
Upper West	12.51	23.33	25.42	38.74	
**Residence**					
Urban	36.07	15.96	44.03	3.94	***
Rural	3.59	23.85	52.93	19.63	
**Rent agreement**					
Owning	13.50	24.61	45.28	16.61	***
Renting	35.73	14.24	45.12	4.91	
Rent free	20.23	16.75	54.72	8.30	
Perching/squatting	30.10	30.06	31.02	8.82	

Source: Authors’ computation from GLSS7

^***^p < 0.01; Row percentages are used; Total percentages are rounded as 100

**Table 3:** Effect of financial inclusion on the choice of solid waste disposal method among households in Ghana

(1)				(2)		
Variable	Burned by household	Public dump	Dumped indiscriminately	Burned by household	Public dump	Dumped indiscriminately
**Financial inclusion (Ref: No)**						
Yes	−0.222^**^	− 0.261^**^	− 0.519^***^	− 0.142	−0.191^*^	− 0.398^***^
	(0.104)	(0.108)	(0.137)	(0.102)	(0.105)	(0.118)
**Income (in Ghana Cedis)**	−0.00000347	− 0.0000111^***^	− 0.0000119^*^	0.0000139	0.00000477	0.0000124
	(0.00000338)	(0.00000323)	(0.00000617)	(0.0000100)	(0.00000654)	(0.0000118)
**Sex (Ref: Female)**						
Male	0.315^***^	−0.0123	0.362^***^	0.312^***^	−0.0152	0.363^***^
	(0.0734)	(0.0689)	(0.0992)	(0.0731)	(0.0688)	(0.0968)
**Educational qualification (Ref: None)**						
Yes	− 0.367^***^	− 0.361^***^	− 0.523^***^	− 0.362^***^	−0.356^***^	− 0.510^***^
	(0.0936)	(0.0857)	(0.103)	(0.0931)	(0.0860)	(0.105)
**Religion (Ref: No religion)**						
Christian	−0.741^***^	− 0.446^***^	− 0.578^***^	− 0.732^***^	−0.437^***^	− 0.571^***^
	(0.207)	(0.163)	(0.199)	(0.207)	(0.162)	(0.198)
Traditionalist/other	−0.321	−0.520^*^	− 0.239	− 0.306	− 0.505	−0.224
	(0.303)	(0.306)	(0.309)	(0.304)	(0.308)	(0.311)
Islam	−1.145^***^	−0.443^**^	− 0.784^***^	− 1.134^***^	−0.431^**^	− 0.779^***^
	(0.262)	(0.204)	(0.251)	(0.259)	(0.203)	(0.249)
**Region (Ref: Western)**						
Central	0.497^**^	−0.213	0.163	0.481^**^	−0.228	0.143
	(0.239)	(0.257)	(0.310)	(0.240)	(0.259)	(0.312)
Greater Accra	−1.069^***^	−2.587^***^	− 1.403^***^	− 1.085^***^	− 2.602^***^	− 1.426^***^
	(0.241)	(0.283)	(0.347)	(0.241)	(0.286)	(0.350)
Volta	0.908^***^	−0.865^***^	0.822^***^	0.895^***^	−0.877^***^	0.806^***^
	(0.261)	(0.287)	(0.296)	(0.262)	(0.288)	(0.298)
Eastern	0.881^***^	−0.299	0.437	0.871^***^	−0.309	0.423
	(0.283)	(0.288)	(0.333)	(0.283)	(0.289)	(0.334)
Ashanti	−0.662^**^	−1.196^***^	−1.038^***^	−0.686^**^	− 1.217^***^	− 1.078^***^
	(0.280)	(0.276)	(0.335)	(0.275)	(0.278)	(0.325)
Brong-Ahafo	0.978^***^	0.430	1.223^***^	0.966^***^	0.420	1.208^***^
	(0.300)	(0.291)	(0.330)	(0.300)	(0.292)	(0.330)
Northern	0.484	−0.649^*^	0.923^**^	0.480	−0.654^*^	0.925^**^
	(0.352)	(0.355)	(0.396)	(0.352)	(0.356)	(0.397)
Upper East	0.716^**^	−2.211^***^	0.449	0.702^**^	−2.223^***^	0.435
	(0.297)	(0.330)	(0.314)	(0.295)	(0.331)	(0.314)
Upper West	−0.261	−1.757^***^	0.232	− 0.274	− 1.770^***^	0.224
	(0.278)	(0.327)	(0.313)	(0.276)	(0.327)	(0.313)
**Residence (Ref: Urban)**						
Rural	1.063^***^	1.229^***^	1.648^***^	1.070^***^	1.235^***^	1.658^***^
	(0.171)	(0.184)	(0.200)	(0.170)	(0.184)	(0.197)
**Age (in years)**	−0.0126^***^	− 0.00513^**^	− 0.0122^***^	− 0.0125^***^	− 0.00507^**^	−0.0121^***^
	(0.00266)	(0.00256)	(0.00391)	(0.00270)	(0.00257)	(0.00393)
**Household size**	0.0128	0.0283^*^	0.0199	0.0116	0.0272	0.0177
	(0.0181)	(0.0170)	(0.0200)	(0.0183)	(0.0171)	(0.0201)
**Rent agreement (Ref: Owning)**						
Renting	−0.643^***^	−0.227^*^	− 0.437^***^	−0.644^***^	− 0.228^*^	−0.429^***^
	(0.129)	(0.116)	(0.131)	(0.129)	(0.116)	(0.134)
Rent free	−0.327^***^	0.123	−0.186	− 0.325^***^	0.124	−0.183
	(0.119)	(0.111)	(0.127)	(0.119)	(0.111)	(0.127)
Perching/squatting	0.140	−0.253	− 0.771	0.148	−0.248	− 0.758
	(0.454)	(0.376)	(0.480)	(0.455)	(0.377)	(0.480)
Financial inclusion*Income						
Yes				−0.0000199^*^	− 0.0000181^**^	− 0.0000329^**^
				(0.0000107)	(0.00000710)	(0.0000131)
Constant	1.581^***^	2.552^***^	0.748^**^	1.528^***^	2.504^***^	0.675^*^
	(0.296)	(0.279)	(0.350)	(0.287)	(0.281)	(0.347)
Observations	9743			9743		

Source: Authors’ computation from GLSS7

Standard errors in parentheses; ^*^ *p* < 0.1, ^**^ *p* < 0.05, ^***^ *p* < 0.01; Reference category for waste disposal method is collected

### Socio-demographic characteristics of respondents and bivariate analysis

In Table 1, we find that as regards the choice of method for households' disposal of solid waste, 47.96% of them use public dump, followed by collected (21.72%), burning (19.45%) and indiscriminate dumping (10.87%). It is worrying to note that only few of the households use the collection method of solid waste disposal.

Concerning the main independent variable; financial inclusion, we find that 55.99% of the household heads have a form of financial inclusion, while 44.01% do not have. Also, majority of the household heads are males (66.62%) and have some form of formal educational qualification (69.75%). Statistics of the remaining socio-demographic variables can be found in Table 1.

In Table 2, as regards the bivariate analysis, the Pearson Chi2 results show that there are statistically significant associations between all the categorical independent variables (financial inclusion, region, religion, educational qualification, sex, residence type and rent agreement) and the choice of solid waste disposal method, all at 1% level of significance. These therefore necessitate a multivariate analysis to find out the effect of financial inclusion on the choice of households' solid waste disposal method, while controlling for other factors since the bivariate analysis takes care of only two variables at a time. The percentage distributions of categorical independent variables with regard to solid waste disposal method, can be found in Table 2.

### Regression analysis of the effect of financial inclusion on the choice of solid waste disposal method

The multinomial probit results of the effect of financial inclusion on the choice of solid waste disposal method are presented in this sub-section (Table 3, (1)). Moreover, we present results of  how financial inclusion affects the choice of solid waste disposal method through a rise in income (Table 3, (2)).

In Table 3 (model without the interaction term (1)), we find that households whose heads are financially included are less likely to choose burning (Coefficient: − 0.22, *p* < 0.05), public dumping (Coefficient: − 0.26, *p* < 0.05) and indiscriminate dumping (Coefficient: − 0.52, *p* < 0.01) of solid waste over the collection method, relative to households whose heads don’t have any form of financial inclusion.

We also find that male-headed households are more likely to choose burning (Coefficient: 0.32, *p* < 0.01) and indiscriminate (Coefficient: 0.36, *p* < 0.01) waste disposal over the collection method, relative to female-headed households. Also, households whose heads have formal educational qualification are less likely to choose burning (Coefficient: − 0.37, *p* < 0.01), public dumping (Coefficient: − 0.36, *p* < 0.01) and indiscriminate dumping (Coefficient: − 0.52, *p* < 0.01) of solid waste over the collection method, as compared with households headed by people without any formal educational qualification (Table 3, (1)).

Also, concerning religion, Christian-headed households are less likely to opt for burning (Coefficient: − 0.74, *p* < 0.01), public dumping (Coefficient: − 0.45, *p* < 0.01) and indiscriminate dumping (Coefficient: − 0.58, *p* < 0.01) of solid waste over the collection method, relative to households whose heads don’t belong to any religion. Similarly, Muslim-headed households are found to be less probable to choose burning (Coefficient: − 1.15, *p* < 0.01), public dumping (Coefficient: − 0.44, *p* < 0.05) and indiscriminate dumping (Coefficient: − 0.78, *p* < 0.01) of solid waste over the collection method, as compared with households whose heads don’t belong to any religious faith. Also, households whose heads belong to the traditional or other faiths are less likely to choose public dumping of solid waste (Coefficient: − 0.52, *p* < 0.1) over the collection method relative to those whose heads are without any religious faith (Table 3, (1)).

The region of residence is also found to play a significant role with regard to the choice of solid waste disposal method. Households in the Central (Coefficient: 0.50, *p* < 0.05), Volta (Coefficient: 0.91, *p* < 0.01), Eastern (Coefficient: 0.88, *p* < 0.01), Brong Ahafo (Coefficient: 0.98, *p* < 0.01) and Upper East (Coefficient: 0.72, *p* < 0.05) regions are more likely to burn than opt for collection method of solid waste disposal, as compared with those in the Western region. Nonetheless, households in the Greater Accra region are less likely to choose burning (Coefficient: − 1.07, *p* < 0.01), public dumping (Coefficient: − 2.59, *p* < 0.01) and indiscriminate dumping (Coefficient: − 1.40, *p* < 0.01) of solid waste over the collection method, relative to those in the Western region. Similar results are found among households in the Ashanti region relative to those in the Western region. Further, rural households are found to be more likely to burn (Coefficient: 1.06, *p* < 0.01), publicly (Coefficient: 1.23, *p* < 0.01) and indiscriminately (Coefficient: 1.65, *p* < 0.01) dump their solid wastes than opt for the collection method, relative to urban households (Table 3, (1)).

As expected, total household wage income (income) is found to be linked with a decrease in the likelihood of public (Coefficient: − 0.00001, *p* < 0.01) and indiscriminate (Coefficient: − 0.00001, *p* < 0.1) dumping of solid waste relative to the collection method. Similarly, rising age of the household head is found to be associated with a decrease in the likelihood of burning (Coefficient: − 0.01, *p* < 0.01), public dumping (Coefficient: − 0.005, *p* < 0.05) and indiscriminate dumping (Coefficient: − 0.01, *p* < 0.01) of solid waste relative to the collection method. Conversely, rising household size is found to be associated with a higher likelihood of resorting to public dumping (Coefficient: 0.03, *p* < 0.1) relative to the collection method. We find those who rent to be less likely to opt for burning (Coefficient: − 0.64, *p* < 0.01), public dumping (Coefficient: − 0.23, *p* < 0.1) and indiscriminate dumping (Coefficient: − 0.44, *p* < 0.01) of solid waste over the collection method, relative to those who own their dwellings. Similarly, those in rent free dwellings are found to be less likely to opt for burning of solid waste (Coefficient: − 0.33, *p* < 0.01) over the collection method, relative to those who own their dwellings (Table 3, (1)).

Regarding the interaction term model (Table 3, (2)), we still find financial inclusion to be associated with a lesser likelihood of choosing public (Coefficient: − 0.19, *p* < 0.1) and indiscriminate (Coefficient: − 0.40, *p* < 0.01) dumping of solid waste relative to the collection method. As regards the interaction between financial inclusion and total household wage income, we find it to be associated with a lesser likelihood of opting for burning (Coefficient: − 0.00002, *p* < 0.1) as well as  public (Coefficient: − 0.00002, *p* < 0.05) and indiscriminate (Coefficient: − 0.00003, *p* < 0.05) dumping of solid waste over the collection method.

The results of majority of the other variables in the model with the interaction term are not qualitatively different from those in the model without the interaction term.

## Discussion

This study investigates the effect of financial inclusion on the choice of solid waste disposal method among households in Ghana. The findings indicate that financial inclusion is associated with a decrease in the likelihood of using burning, public dumping and indiscriminate dumping as means of solid waste disposal relative to the collection method. Thus, financial inclusion helps households to adopt a healthier or proper form of waste disposal.

The outcome of the effect of financial inclusion is not farfetched because, studies have revealed financial inclusion to enhance poverty alleviation, economic growth and net wealth benefits [22–25], hence, boosting the ability of people to afford healthy solid waste disposal methods which normally come at a fee. In Ghana, financial inclusion has been found to be negatively associated with open defecation and sharing of toilet facilities among households [33]. Similarly, financial inclusion has been found to increase access to basic drinking water and sanitation in Africa [34]. Hence, given the role of financial inclusion in enhancing demand for health inputs (such as basic drinking water and sanitation, healthy solid waste disposal), it is not surprising that it (financial inclusion) has been found by Immurana et al. [35] to enhance population health. Moreover, unsurprisingly, both income (in the model without the interaction term) and the interaction of financial inclusion with income are found to be associated with the adoption of a healthy solid waste disposal method. The finding on the role of income in adopting healthy solid waste disposal is in tandem with studies by Alhassan et al. [9], Boateng et al. [13] and Awunyor-Vitor et al. [21] in Ghana.

Theoretically, our findings on financial inclusion and income corroborate the postulation of the theory of demand for health that, as income increases, the demand for health inputs (such as healthy solid waste disposal) also increases (see Grossman, [27]). Moreover, since the poor are more likely to be unable to afford the fees associated with healthy solid waste disposal methods, our findings provide support for the vulnerable group theory of financial inclusion which according to Ozili [28] states that, programmes aimed at enhancing financial inclusion should be focused on vulnerable groups such as the poor, the elderly and women. Thus, to enhance the choice of healthy solid waste disposal methods, improving financial inclusion among poor people, could be a very useful strategy.

The sex of household head is also found to have a statistically significant association with  the choice of solid waste disposal method. The finding on male-headed households being more likely to choose unhealthy waste disposal methods (burning and indiscriminate waste disposal) compared with female-headed households could be attributed to males not caring much about domestic activities such as waste disposal, sweeping, washing and cleaning relative to females. Thus, in a typical Ghanaian setting, such domestic activities are handled by females in the household. Also, the role of the educational qualification of the household head concerning the less likelihood of choosing unhealthy means of solid waste disposal is not surprising. This is because, education, through topics on sanitation and hygiene can make individuals to better understand the importance of healthy waste disposal methods such as collection, as compared with less healthy ones (burning, public dumping and indiscriminate dumping). In fact, education has been revealed to be positively associated with the demand for (or access to) even other health enhancing goods and services [36–42]. The findings on male-headed households and education with regard to healthy method of waste disposal are similar to the results of Adzawla et al. [8], Addai and Danso-Abbeam [7] and Alhassan et al. [9].

Religion is also found to have a statistically significant association with the choice of solid waste disposal method among households. Thus, since Christians and Muslims place greater emphasis on cleanliness or hygiene as a religious practice, they are more likely to be  willing to adopt healthy forms of waste disposal relative to those with no religion.

The findings on households in the Central, Volta, Eastern, Brong Ahafo and Upper East regions being more likely to opt for unhealthy (burning) method of solid waste disposal, as compared with those in the Western region, concur with Adzawla et al. [8]. Nonetheless, the outcomes of Greater Accra and Ashanti regions being less likely to choose unhealthy means of solid waste disposal could be due to their more urbanised nature which makes them more likely to have access to firms and institutions who provide waste management services [12]. This is confirmed by our study because rural households are found to be more likely to adopt less healthy means of solid waste disposal relative to urban households. Indeed, Boateng et al. [12] found more urban communities to use the collection method of waste disposal relative to rural communities. Also, Kodua and Anaman [16] found urban households to be less likely to resort to open dumping of waste.

Rising age of the household head is also found to increase the likelihood of adopting a healthy waste disposal method. This outcome is not surprising as the elderly might be more experienced as regards the importance of disposing waste properly relative to the younger ones. The finding is similar to those of Adzawla et al. [8] and Alhassan et al. [9]. Last but not the least, the higher likelihood of households who rent to adopt a healthy waste disposal method relative to those who own their dwellings could be that, households who rent are forced to adopt disposing solid waste by collection in order to avoid harming or disturbing other tenants through burning or indiscriminate dumping of solid waste.

## Conclusion

A number of countries in the world including Ghana, are trying to find best means of managing the huge amount of solid waste generated by their residents. This is because, improper disposal of solid waste has numerous negative health and economic implications. To this end, a number of studies on Ghana have examined issues of solid waste disposal methods. Notwithstanding, while financial inclusion is deemed as an enabler of proper waste disposal, none of these studies considered it. This study therefore investigates the effect of financial inclusion on the choice of solid waste disposal method among households in Ghana, using data from the GLSS7. We employ the multinomial probit regression as the estimation technique while controlling for factors such as education, sex, age, religion, region, residence type (urban/rural), household size, total household wage income (income) and rent agreement. Our findings show that financial inclusion increases the likelihood of households opting for the collection (healthy) method of solid waste disposal relative to burning, public dumping and indiscriminate disposal of solid waste. Further, a major channel via which financial inclusion enhances the choice of healthy solid waste disposal method is through income.

The conclusion is that, financial inclusion enables households to opt for a healthy solid waste disposal method, especially through an enhancement in income. Thus, to ensure the adoption of healthy solid waste disposal methods in Ghana, deliberate effort should be made towards making financial products and services, easily and cheaply available to people, especially the poor. Doing so would provide them with higher income, hence boosting their ability to pay for healthy solid waste disposal.

## Data Availability

The de-identified data used for the study is publicly available (open access) from the website of the Ghana Statistical Service (https://www2.statsghana.gov.gh/nada/index.php/catalog/97/get_microdata) after creating a free account. No permission or approval is required to access the data. Further details about the data can be obtained by contacting, Ghana Statistical Service, Head Office, P.O. Box GP 1098, Accra-Ghana, E-mail: info@statsghana.gov.gh.
